# Phenotypic and Functional Characterization of Human Memory T Cell Responses to *Burkholderia pseudomallei*

**DOI:** 10.1371/journal.pntd.0000407

**Published:** 2009-04-07

**Authors:** Patcharaporn Tippayawat, Wipawee Saenwongsa, Jirawan Mahawantung, Duangchan Suwannasaen, Ploenchan Chetchotisakd, Direk Limmathurotsakul, Sharon J. Peacock, Philip L. Felgner, Helen S. Atkins, Richard W. Titball, Gregory J. Bancroft, Ganjana Lertmemongkolchai

**Affiliations:** 1 The Centre for Research & Development of Medical Diagnostic Laboratories, Faculty of Associated Medical Sciences, Khon Kaen University, Khon Kaen, Thailand; 2 Department of Medicine, Faculty of Medicine, Khon Kaen University, Khon Kaen, Thailand; 3 Mahidol-Oxford Tropical Medicine Research Unit, Faculty of Tropical Medicine, Mahidol University, Bangkok, Thailand; 4 Department of Medicine/Division of Infectious Diseases, University of California Irvine, Irvine, California, United States of America; 5 Defence Science and Technology Laboratory, Porton Down, United Kingdom; 6 School of Biosciences, University of Exeter, Exeter, United Kingdom; 7 Immunology Unit, London School of Hygiene and Tropical Medicine, London, United Kingdom; Weill Medical College of Cornell University, United States of America

## Abstract

**Background:**

Infection with the Gram-negative bacterium *Burkholderia pseudomallei* is an important cause of community-acquired lethal sepsis in endemic regions in southeast Asia and northern Australia and is increasingly reported in other tropical areas. In animal models, production of interferon-gamma (IFN-γ) is critical for resistance, but in humans the characteristics of IFN-γ production and the bacterial antigens that are recognized by the cell-mediated immune response have not been defined.

**Methods:**

Peripheral blood from 133 healthy individuals who lived in the endemic area and had no history of melioidosis, 60 patients who had recovered from melioidosis, and 31 other patient control subjects were stimulated by whole bacteria or purified bacterial proteins in vitro, and IFN-γ responses were analyzed by ELISPOT and flow cytometry.

**Findings:**

*B. pseudomallei* was a potent activator of human peripheral blood NK cells for innate production of IFN-γ. In addition, healthy individuals with serological evidence of exposure to *B. pseudomallei* and patients recovered from active melioidosis developed CD4^+^ (and CD8^+^) T cells that recognized whole bacteria and purified proteins LolC, OppA, and PotF, members of the *B. pseudomallei* ABC transporter family. This response was primarily mediated by terminally differentiated T cells of the effector–memory (T_EMRA_) phenotype and correlated with the titer of anti-*B. pseudomallei* antibodies in the serum.

**Conclusions:**

Individuals living in a melioidosis-endemic region show clear evidence of T cell priming for the ability to make IFN-γ that correlates with their serological status. The ability to detect T cell responses to defined *B. pseudomallei* proteins in large numbers of individuals now provides the opportunity to screen candidate antigens for inclusion in protein or polysaccharide–conjugate subunit vaccines against this important but neglected disease.

## Author Summary

The Gram-negative bacterium, *Burkholderia pseudomallei*, is a public health problem in southeast Asia and northern Australia and a Centers for Disease Control and Prevention listed Category B potential bioterrorism agent. It is the causative agent of melioidosis, and clinical manifestations vary from acute sepsis to chronic localized and latent infection, which can reactivate decades later. *B. pseudomallei* is the major cause of community-acquired pneumonia and septicemia in northeast Thailand. In spite of the medical importance of *B. pseudomallei*, little is known about the mechanisms of pathogenicity and the immunological pathways of host defense. There is no available vaccine, and the mortality rate in acute cases can exceed 40% with 10–15% of survivors relapsing or being reinfected despite prolonged and complete treatments. In this article, we describe cell-mediated immune responses to *B. pseudomallei* in humans living in northeast Thailand and demonstrate clear evidence of T cell priming in healthy seropositive individuals and patients who recovered from melioidosis. This is the most detailed study yet performed on the cell types that produce interferon-gamma to *B. pseudomallei* in humans and the antigens that they recognize and the first to study large sample numbers in the primary endemic focus of melioidosis in the world.

## Introduction

Melioidosis is a serious infectious disease in Southeast Asia and Northern Australia caused by the soil-dwelling Gram-negative bacterium, *Burkholderia pseudomallei* [1]. In Northeast Thailand, the mortality rate for acute melioidosis remains high, approximately 50%, despite recent advances in antibiotic treatments. Serological evidence, based on the indirect hemagglutination assay (IHA), suggests that 80% of people living in endemic areas have been exposed to *B. pseudomallei*, without showing clinical symptoms [1]–[3]. Recurrent melioidosis can also occur either as relapse after antibiotic treatment or re-infection [3],[4]. *B. pseudomallei* is classified as a NIAID category B potential agent for biological terrorism [5]. The mechanism that enables the organism to avoid the bactericidal effects of the host immune response has never been fully understood, and there are no licensed vaccines.

*B. pseudomallei* is able to disseminate throughout the body, invades non-phagocytic cells and replicates in phagocytes [6],[7]. In mice, *B. pseudomallei* is a potent inducer of IFN-γ and IFN-γ inducing cytokines such as IL-12, IL-18 and TNF *in vitro* and IFN-γ is essential for resistance *in vivo* via the activation of macrophages for both oxygen dependent and independent killing mechanisms [8]. In mice, NK cells and bystander CD8^+^ T cells provide innate production of IFN-γ [9], while IFN-γ secreting, antigen-specific CD4^+^ T cells contribute to protection against primary infection with *B. pseudomallei* and following immunization with experimental vaccines in vivo [10],[11]. In addition, murine models of vaccination with dendritic cells pulsed with heat killed *B. pseudomallei* in the presence of CpG oligodeoxynucleotides showed significant levels of protection [12] suggesting the role of specific T cells in host protection.

In contrast, the mechanisms of cell-mediated immunity to *B. pseudomallei* in humans are poorly understood. IFN-γ, IL-12, IL-18 and TNF are found in plasma samples from acute, septic melioidosis but the IFN-γ producing cells have not been well characterized [13]. Previous studies in small numbers of patients in northern Australia and Papua New Guinea who recovered from melioidosis have demonstrated evidence of T cell priming to *B. pseudomallei*, but the characteristics of the responding cell populations and the antigens recognized have not been defined [14],[15].

Here, we analyzed a large cohort of individuals from the melioidosis endemic region of Thailand to identify the cellular sources of IFN-γ in response to whole *B. pseudomallei* and the bacterial ABC transporter proteins LolC, OppA and PotF which are T cell immunogens in mice and candidate vaccine antigens [16],[17]. Peripheral blood cells from healthy individuals with serological evidence of exposure to *B. pseudomallei* and recovered melioidosis patients (but not seronegative control subjects) showed evidence of CD4 and CD8 T cell priming to both whole bacteria and purified *B. pseudomallei* antigens. Together with a prominent IFN-γ response from NK cells, these sources of IFN-γ may contribute to host resistance against melioidosis in the endemic setting.

## Materials and Methods

The study and the consent forms were approved by the Khon Kaen University Ethics Committee for Human Research (Project number HE470506). Informed consent was obtained from all the subjects recruited into the study.

### Blood samples

Blood samples from 133 healthy donors who had no clinical history of melioidosis were collected at the Blood Bank, Khon Kaen University, Thailand. Another set of blood samples was obtained from patients and control subjects at Sappasithiprasong Hospital, Thailand for cellular studies by ELISPOT assay. Patients were defined as those who had recovered from melioidosis (previously diagnosed by isolation of *B. pseudomallei* from blood or tissues) and completed antibiotic treatment (n = 36). Non infected control subjects (n = 21) were those who attended the hospital for non infectious reasons at the diabetic clinic and had no history of clinical melioidosis, and were matched for age, sex, occupation, the presence of diabetes as an underlying condition and lived in the same endemic area. In addition, 24 recovered melioidosis patients and 10 healthy control subjects were enrolled, using the same criteria, at Srinagarind Hospital, Thailand for cellular sources of IFN-γ, kinetics and memory cells assayed by flow cytometry. The subjects who had antibodies to *B. pseudomallei* at a titer of 1∶40 or greater by IHA were considered seropositive [15],[18]. None of the subjects had any clinical sign or symptoms of any infection including HIV/AIDS at the time of blood collection.

### *In vitro* cell stimulation

*B. pseudomallei* strain K96243 is a clinical isolate from Thailand and is the prototype genome sequence strain [19]. Whole heat-killed *B. pseudomallei* (hkBp) was prepared by heating the bacteria at 100°C for 20 minutes, washed twice with PBS pH 7.4, aliquoted and stored at −80°C. The number of viable bacteria was determined by colony-forming counts and defined as colony-forming units (CFU) prior to heating. Recombinant *B. pseudomallei* ABC transporter proteins (LolC, OppA and PotF) were prepared as previously described [16],[17] and used as test stimulators in this study. Phytohemagglutinin (PHA) (Biochrom AG, Germany), human recombinant IL-12, and IL-15 (BD Biosciences, USA) and a MHC class I-restricted T cell epitope control of pooled peptides of cytomegalovirus, Epstein Barr virus and influenza virus (CEF) were used as positive controls (Mabtech, AB, Sweden). Recombinant protein from *Francisella tularensis*, FT1823 [20] was included as a non related protein/negative control.

### Enzyme-linked immunospot assay (ELISPOT) for human IFN-γ

Peripheral blood mononuclear cells (PBMCs) from each subject were isolated from heparinized blood samples by density centrifugation on Ficoll-Hypaque and adjusted the number of cells as required prior to stimulation. In brief, 96-well PVDF-plates (MSIP, Millipore) were previously coated overnight with 15 µg/ml 1D1K anti-human IFN-γ at 4°C. Fresh PBMCs were added in duplicate wells at 5×10^5^ PBMCs/well and each stimulator was added at the optimal concentration. After 42 hours, secreted IFN-γ was detected by adding 1 µg/ml biotinylated mAb 7-B6-1-biotin for IFN-γ for 3 hours and followed by 1 µg/ml streptavidin-alkaline phosphatase (Mabtech, AB, Sweden) prior to enumeration under a stereomicroscope. The responses were compared in the absence or presence of 0.3 µg/ml cyclosporin A (CsA, Sigma, USA).

### Intracellular cytokine staining assay by flow cytometry

Whole blood samples were firstly analyzed for complete blood count using an automatic machine (Sysmex, Germany). The number of absolute lymphocytes was then adjusted to 9×10^5^ lymphocytes/ml by diluting with completed RPMI medium (10% FBS supplement). The adjusted cells in 100 µl were added into 96 well culture plates and added up by another 100 µl of 2× concentration of stimulators and incubated at 37°C with 5% CO_2_. Cultured cells were blocked with 10 µg/ml brefeldin A (Sigma, USA) for 3 hours prior to the end of the incubation time. Then washed and blocked with anti-CD16 (BD Biosciences). The following antibodies were used for immune cell surface staining: FITC anti-CD4, PE anti-CD8 or PE anti-CD56 (BD Biosciences) and Tricolor anti-CD3 (Invitrogen, USA). In addition, cell surface markers for memory T cell phenotypes were included: FITC anti-CCR7 (R&D systems, USA), PE anti-CD45RA (Invitrogen) and Tricolor anti-CD4 or CD8 (Invitrogen). Isotype-matched control antibodies were used in each analysis. After 30 min of staining, followed by fixation with 10% paraformaldehyde-PBS overnight at room temperature, cells were then permeabilized by 0.12% saponin (Sigma, USA) for 15 min followed by APC anti-IFN-γ (Invitrogen, USA) for 30 min prior to analysis by FACScalibur with CELLQuest software (BD Biosciences, USA).

### Statistical analysis

Statistical analysis (one way-ANOVA, unpaired and paired t-test) was performed using Graphpad Prism version 5 software (GraphPad, San Diego, CA, USA). A *P*-value<0.05 was considered statistically significant.

## Results

### Cellular immune responses to *B. pseudomallei* of healthy individuals living in an endemic area of melioidosis

**Figure 1:**
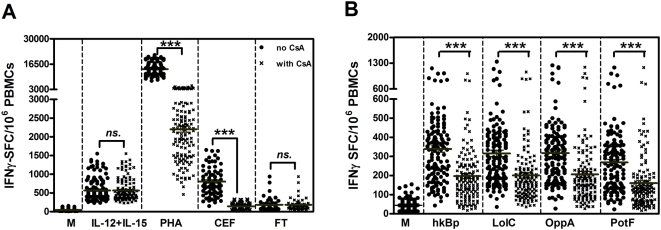
IFN-γ responses to *B. pseudomallei* of 133 healthy individuals living in Northeast Thailand. PBMCs of healthy blood donors were stimulated with whole bacteria, recombinant Bp proteins and control stimulators for 42 hours in vitro and IFN-γ secreting cells were enumerated by ELISPOT. IFN-γ spot forming cells (SFC) per 1×10^6^ PBMCs in response to (A) control stimulators including medium alone (M), cytokines (1 µg/ml IL-12 plus IL-15), 1.25 µg/ml PHA, 2 µg/ml pooled viral peptides (CEF) and 1 µg/ml non related protein of *F. tularensis* (FT) and (B) IFN-γ responses to 3×10^6^ CFU/ml heat killed *B. pseudomallei* (hkBp), 1 µg/ml ABC transporter proteins of *B. pseudomallei* (LolC, OppA and PotF). IFN-γ spots were enumerated in the absence (closed circles) and presence (crossed) of 0.3 µg/ml cyclosporin A (CsA). Horizontal lines represent the mean value of each group, *** *P*<0.0001, ns-non statistical significance (paired t-test).

To examine the cellular immune response to *B. pseudomallei* of healthy individuals living in Northeast Thailand, PBMCs of 133 donors from the Blood Bank at Khon Kaen University, Thailand were cultured with whole bacteria, recombinant *B. pseudomallei* ABC transporter proteins (LolC, OppA and PotF) or control stimulators and 42 hours later assayed for IFN-γ production by ELISPOT. We have previously shown in mice that several different cell types contribute to IFN-γ responses to *B. pseudomallei* in vitro; NK cells and bystander T cells produce IFN-γ indirectly via a cytokine mediated pathway which is not blocked by cyclosporin A (CsA), whereas specific *B. pseudomallei* primed T cells respond via a CsA sensitive T cell receptor (TCR) dependent process [9],[11]. To validate this approach in human peripheral blood, we initially compared the CsA sensitivity of cytokine (IL-12+IL-15), mitogen (PHA) or antigen specific IFN-γ responses in vitro in the presence or absence of CsA. Compared to medium alone controls, cells incubated with PHA or a pooled cocktail of established T cell reactive peptides from pathogens known to be present in the Thai population (CMV, EBV and influenza) showed strong IFN-γ responses which were inhibited in the presence of CsA (Figure 1A; *P*<0.0001, paired t-test). In contrast, the IFN-γ response to IL-12/IL-15 or the low but detectable background response observed in cells incubated with an irrelevant *Francisella tularensis* control protein were not CsA susceptible. Moreover, the results revealed that whole *B. pseudomallei* (hkBp) and three Bp-derived ABC transporter proteins (LolC, OppA and PotF) could induce IFN-γ responses via TCR independent (innate) and dependent (specific) pathways in healthy individuals in vitro (Figure 1B).These IFN-γ responses were in a dose dependent manner ranging between 1×10^4^–1×10^7^ CFU/ml hkBp and 0.1–3.0 µg/ml of the 3 proteins (data not shown).

### Specific T cell responses to *B. pseudomallei* correlate with serological evidence of exposure to the bacterium

**Figure 2:**
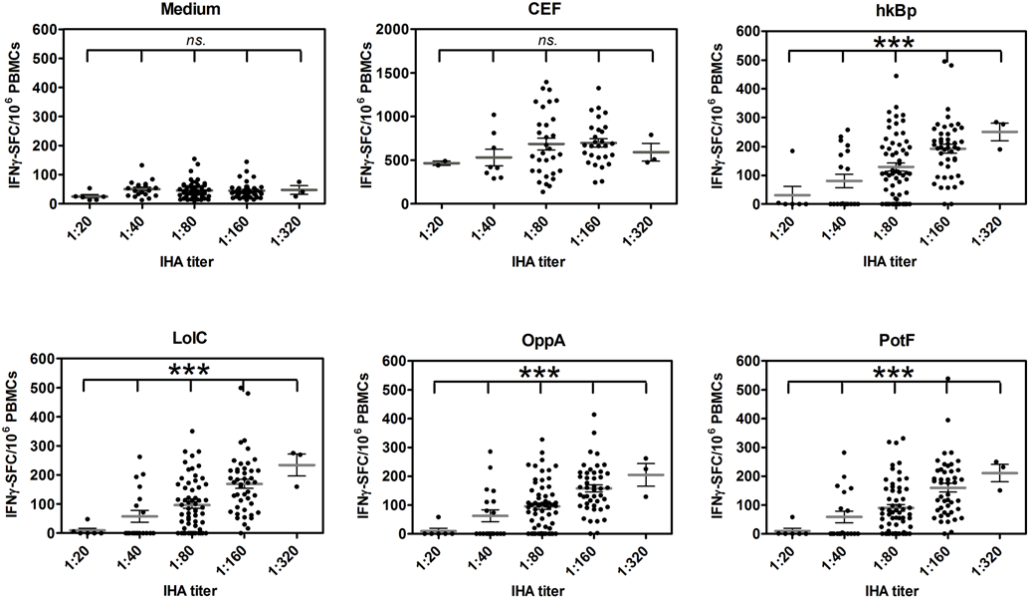
Distribution of specific T cell responses to whole *B. pseudomallei* and three ABC transporter proteins. PBMCs from 133 healthy blood donors as described in Figure 1 were classified according to plasma IHA antibody titers; 1∶20, 1∶40, 1∶80, 1∶160 and 1∶320 (n = 6, 19, 60, 45 and 3, respectively) and analyzed for specific (CsA sensitive) IFN-γ secreting spots assayed by ELISPOT in response to medium alone, 2 µg/ml pool viral peptides (CEF), whole *B. pseudomallei* (hk Bp) and Bp derived ABC transporter proteins (LolC, OppA and PotF). *** *P*<0.0001, ns-non statistical significance (one way ANOVA).

IHA has been widely used as routine serologic test for melioidosis with the threshold titer of 1∶40 in the endemic area indicating previous exposure to *B. pseudomallei* [15],[18]. To investigate whether the magnitude of the cellular immune response correlated with evidence of exposure to *B. pseudomallei* by serology, 133 healthy donors were classified into five groups based on their *B. pseudomallei* antibody IHA titers (as 1∶20, 1∶40, 1∶180, 1∶160 and 1∶320 (n = 6, 19, 60, 45 and 3, respectively; Figure 2). The results revealed that the continual increase of the average values of specific (CsA sensitive) IFN-γ spots in response to *B. pseudomallei* and its proteins was significantly correlated with increasing antibody titers (*P*<0.0001, one way ANOVA). No such correlation was observed in the response to CEF vs. medium controls or for the innate (CsA resistant) IFN-γ spots to *B. pseudomallei* (*P*>0.05, one way ANOVA). Thus environmental exposure to *B. pseudomallei* in the endemic region of NE Thailand generates both T cell and B cell responses to *B. pseudomallei* in healthy individuals even in the absence of disease.

### Cellular immune responses to *B. pseudomallei* in patients recovered from melioidosis

**Figure 3:**
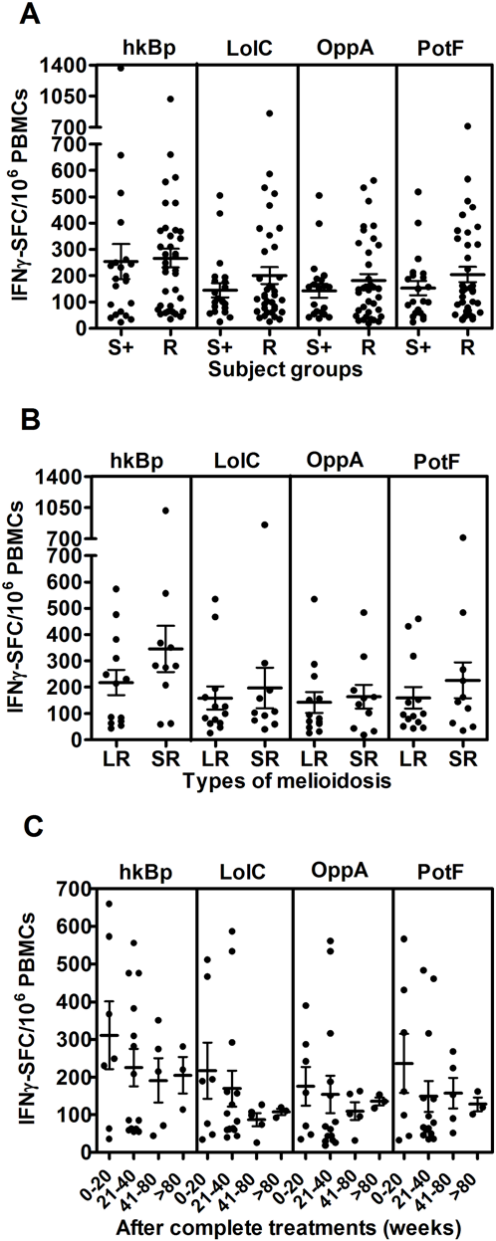
Specific T cell responses to *B. pseudomallei* of recovered melioidosis cases vs. seropositive control subjects. (A) Specific (CsA sensitive) IFN-γ secreting spots in response to medium alone, whole *B. pseudomallei* and Bp-derived ABC transporter proteins, LolC, OppA and PotF were analyzed from 36 recovered melioidosis cases (R) and 21 seropositive control subjects (S+). (B) Of these 36 recovered melioidosis cases, only 24 cases were analyzed according to the previous history of localized melioidosis (LR, n = 13) and septicemic melioidosis (SR, n = 11) and (C) the time of sample collection (weeks) after completion of antibiotic treatments of recovered melioidosis cases. Horizontal lines represent mean±SE value of each group. Medium alone of recovered melioidosis and seropositive control groups was less than 5 IFN-γ SFC/10^6^ PBMCs.

To assess the extent of T cell priming in patients who had survived active infection, specific T cell responses to whole bacteria and recombinant proteins of *B. pseudomallei* were studied in 36 recovered melioidosis cases and 21 other patient control subjects from the same endemic region chosen on the basis of same age, sex and occupation with no history of clinical melioidosis but who were seropositive for *B. pseudomallei* exposure. The frequency of IFN-γ producing cells was significantly increased in recovered patients compared to seronegative control subjects (data not shown) but was similar to that observed in seropositive healthy individuals (Figure 3A) (*P*>0.05, unpaired t-test). However, there were some individuals who had no specific IFN-γ producing cells to *B. pseudomallei* above the background of medium control in both groups. Of note, IFN-γ levels as quantified by ELISA were significantly higher in recovered melioidosis patients than seropositive individuals (Figure S1). According to Figure 3B, the comparison of IFN-γ responses between patients who recovered from melioidosis with a history of localized infection (n = 13) and severe sepsis (n = 11) did not show any significant difference. These specific T cell responses declined over the time but remained detectable after 80 weeks (Figure 3C). These results indicated that either whole *B. pseudomallei* or its proteins could trigger the cellular immune response following re-exposure to the microorganism in vitro up to 80 weeks post admission.

Diabetes mellitus (DM) is a major risk factor for human melioidosis [21], and only 4 cases of recovered melioidosis without DM were found in this study. Even though we observed no difference between IFN-γ responses of these groups, it remains inconclusive for the effect of diabetic condition on host T cell responses (data not shown). In addition, recovered melioidosis patients with a history of recurrent infection (n = 6) compared to those with a single disease episode (n = 30) also showed no statistically significant difference (data not shown); suggesting that under these conditions IFN-γ responses do not differentiate between primary and recurrent melioidosis.

### Cellular sources and kinetics of IFN-γ in response to B. *pseudomallei*

**Figure 4:**
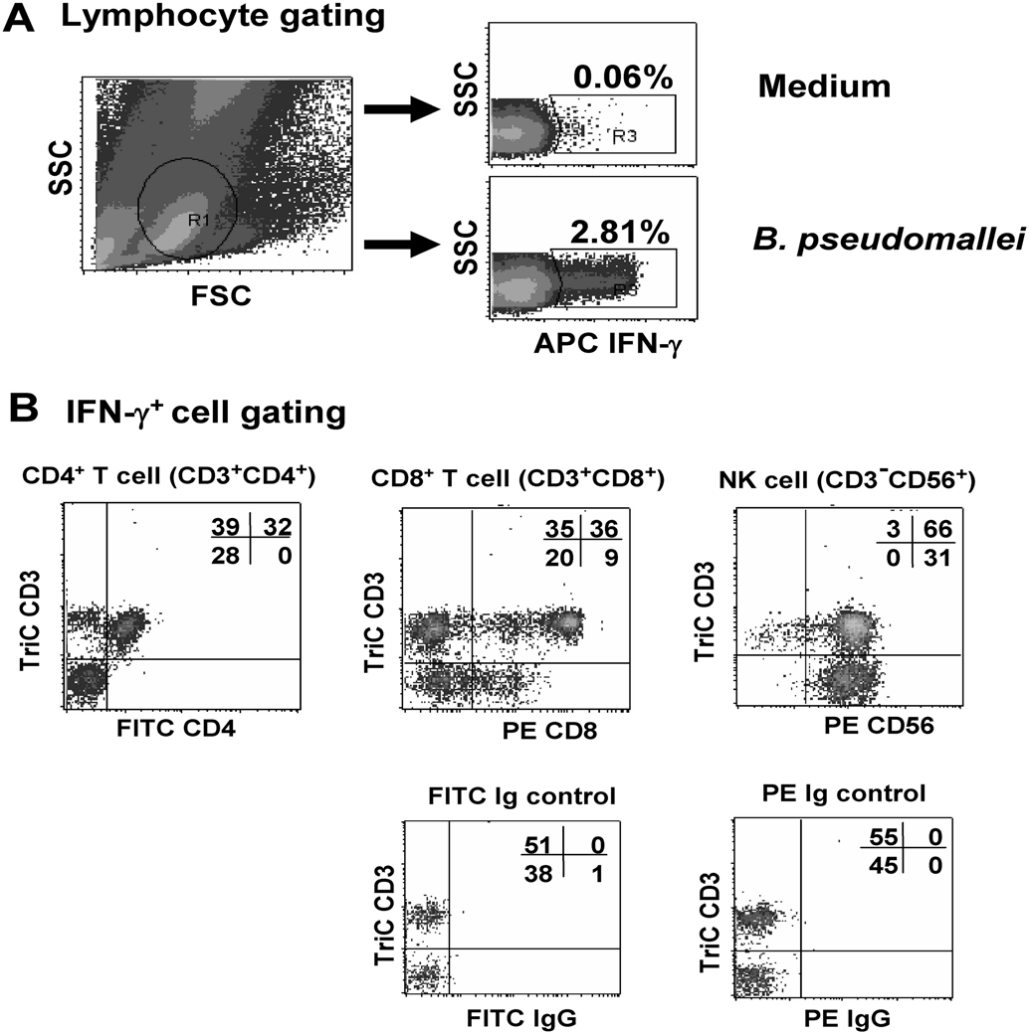
Identification of IFN-γ secreting T cells responding to whole *B. pseudomallei* by four-color flow cytometry. Whole blood samples from eight recovered melioidosis cases and six seropositive control subjects were incubated with whole *B. pseudomallei* for 12 hours and stained for intracellular IFN-γ vs. three immune cell surface markers (Tri-color anti-CD3, FITC-anti-CD4 and PE-anti-CD8 or PE-anti-CD56). The profile from one representative donor of seropositive control subjects, (A) gated on lymphocyte cells, and (B) gated on IFN-γ^+^ cells.

To identify the cellular sources of IFN-γ responses to *B. pseudomallei*, whole blood samples from six seropositive healthy individuals and ten recovered melioidosis cases (all with IHA antibodies 1∶40 or greater) were restimulated with *B. pseudomallei* in the absence of CsA and analyzed by four-color flow cytometry. As shown from one representative of seropositive group, the small lymphocyte area was gated (Figure 4A) and analysis of IFN-γ^+^ cells showed that NK cells (CD3^−^CD56^+^), CD4^+^ T (CD3^+^CD4^+^) and CD8^+^ T (CD3^+^CD8^+^) cells all contributed to IFN-γ production to hkBp (Figure 4B). A dominant contribution of CD4^+^ and CD8^+^ T cells on IFN-γ production was confirmed by significant reduction of specific IFN-γ (CsA sensitive) spots following depletion of CD3, CD4 and/or CD8 cells (Figure S2). In addition, the mean fluorescent intensities (MFI) of intracellular IFN-gamma staining of CD3^+^ and CD3^−^ (NK) cells of 4 recovered melioidosis cases were analyzed and revealed that the average MFI of IFN-gamma gated on CD3^+^ cells was significantly higher than CD3^−^ (NK) cells (*P*<0.05, paired t-test) (Figure S3). Together with our finding that the ELISPOT size of remaining IFN-γ^+^ cells after T cell depletion was very small suggests that innate (CsA resistant) cells produce less of this cytokine compared to specific T cells.

**Figure 5:**
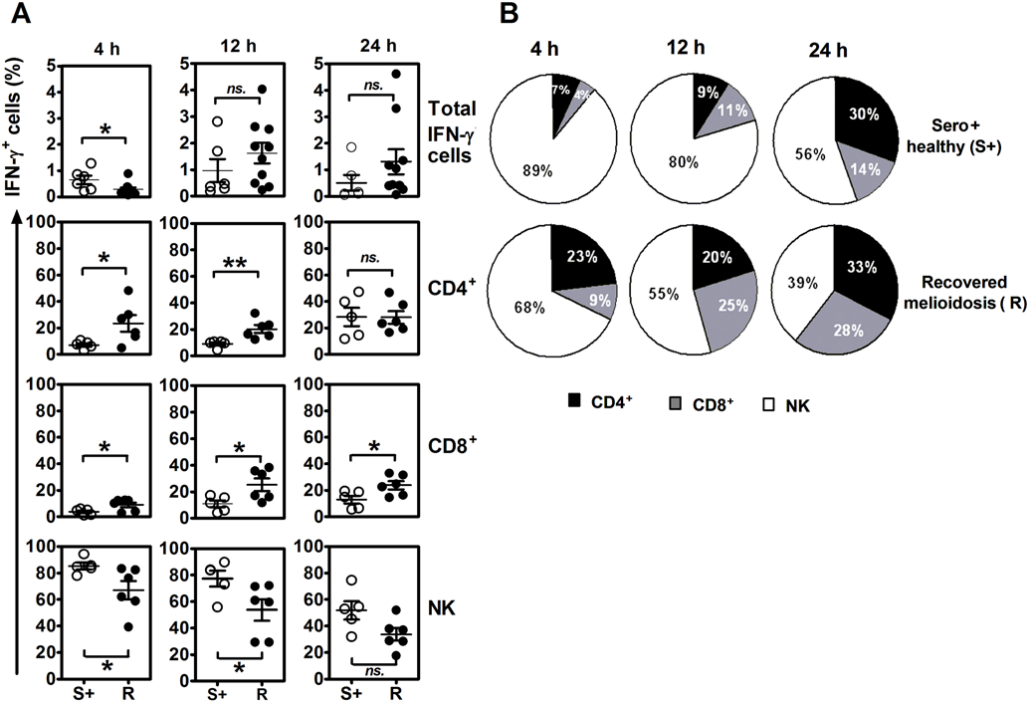
Kinetics of IFN-γ producing cells from ten recovered melioidosis cases and six seropositive control subjects in response to *B. pseudomallei* in vitro. Whole blood samples were incubated with whole *B. pseudomallei* for 4, 12 and 24 hours and stained for intracellular IFN- γ vs. three immune cell surface markers (Tri-color anti-CD3, FITC-anti-CD4 and PE-anti-CD8 or PE-anti-CD56). (A) Distribution of individual responses, horizontal lines indicate mean±SE values of the group and (B) the relative contribution of CD4, CD8 and NK cells to produce IFN- γ of ten recovered melioidosis cases (R) vs. six seropositive control subjects (S+), gated on IFN-γ^+^ cells. * *P*<0.05, ** *P*<0.005, ns-non significant (unpaired t-test).

The analysis of blood samples from 6 seropositive individuals and 10 recovered melioidosis cases showed background staining of total IFN-γ producing cells in medium alone at 0.03±0.01 and 0.3±0.09% (mean±SE), respectively. The relative contribution of each cell type to the IFN-γ response to *B. pseudomallei* also varied according to the time point examined in culture after addition of the bacteria. NK cells appeared to respond more rapidly than T cells and significantly contributed to the production of rapid IFN-γ at 4 hours and decreased over time in both groups (Figure 5). On the one hand, the frequency of IFN-γ producing NK cells were significantly higher in seropositive healthy individuals than recovered melioidosis at 4 and 12 hours (*P*<0.05, unpaired t-test). On the other hand, there was a statistically significant difference of IFN-γ producing CD4^+^ T cells being higher in recovered melioidosis than the seropositive individuals at both time points and all three time points for IFN-γ producing CD8^+^ T cells. These results demonstrated the increasing contribution to IFN-γ production over the time from T cell subsets, particularly in the recovered melioidosis group.

### Rapid responses of memory phenotype T cells to whole *B. pseudomallei*

**Figure 6:**
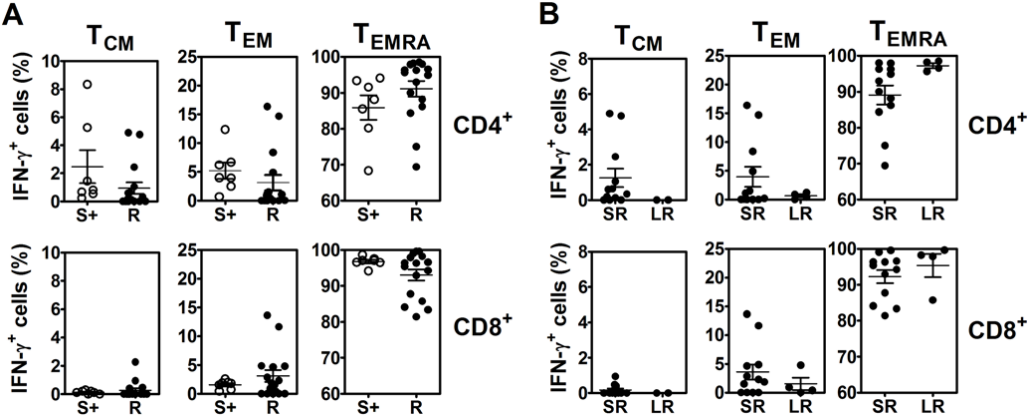
Memory phenotypes of IFN-γ producing cells from recovered melioidosis cases vs. seropositive healthy control subjects in response to *B. pseudomallei* in vitro. Whole blood samples were incubated with whole *B. pseudomallei* for 12 hours and stained for intracellular IFN- γ vs. three immune cell surface markers (Tri-color anti-CD4 or CD8, FITC-anti-CCR7 and PE-anti-CD45RA), gated on IFN-γ^+^ cells. The distribution of memory phenotype subsets i.e., central memory (T_CM_, CD45RA^−^CCR7^+^), effector memory (T_EM_, CD45RA-CCR7^−^) and terminally differentiated T effector memory (T_EMRA_, CD45RA^+^CCR7^−^) of (A) 16 recovered melioidosis (R) vs. 7 seropositive individuals (S+), (B) recovered melioidosis with the history of septicemic (SR, n = 12) vs. localized (LR, n = 4) melioidosis. Horizontal lines indicate mean±SE values of the group.

To investigate whether the rapid IFN-γ producing T cells in response to *B. pseudomallei* were memory T-cell phenotypes, immune subsets of human memory T cells were identified based on the cell surface expression of CD45RA and CCR7 [22] in 16 recovered melioidosis cases and 7 seropositive control subjects. The results demonstrated the percentages of IFN-γ producing memory T cells and the majority of memory CD4^+^ and CD8^+^ T cells of both groups were revealed as terminally differentiated T effector memory (T_EMRA_) cells which was significantly higher than the other memory phenotypes of effector memory (T_EM_) and central memory (T_CM_) cells of CD4^+^ and CD8^+^ T cells (*P*<0.0001, unpaired t-test) (Figure 6A).

When the clinical histories of these 16 recovered melioidosis subjects were analyzed, distinctive patterns of memory T cell phenotypes were revealed. The memory T cells of the septicemic melioidosis group (n = 12) were T_EMRA_ significantly greater than T_EM_ and T_CM_ of both CD4^+^ and CD8^+^ subsets (*P*<0.0001, unpaired t-test). Interestingly, there was a trend of localized melioidosis group (n = 4) showed stronger responses of T_EMRA_ with small contribution of T_CM_ cells and T_EM_ cells, but it was not statistically significant (Figure 6B).

## Discussion

*Burkholderia pseudomallei* is an important cause of community acquired sepsis and death in endemic regions of SE Asia and Northern Australia and is listed as potential bioterrorism threat. Yet despite its current and potential impact on public health our understanding of immune defenses against this pathogen are incomplete. *B. pseudomallei* is capable of extensive extracellular growth and abscess formation, but is also genetically adapted to survive and replicate within host cells [6],[23]. It is killed by IFN-γ activated macrophages in vitro [24], making cell mediated immunity a potentially important component of resistance. Here, a total of 224 individuals living in the endemic area of NE Thailand of varying immunological and clinical history for exposure to *B. pseudomallei* were examined for the magnitude and characteristics of their IFN-γ responses following restimulation of whole blood with whole bacteria or *B. pseudomallei* derived antigens in vitro.

Northeast Thailand is the primary endemic focus of melioidosis in SE Asia and the majority of individuals show evidence of seroconversion from an early age [2]. To obtain an initial estimate of the diversity of the IFN-γ response in this setting, blood samples from 133 randomly selected individuals who had no clinical history of melioidosis were tested for reactivity to *B. pseudomallei* by IFN-γ ELISPOT. The majority showed clear induction of IFN-γ positive cells above that of medium alone controls in the presence of whole bacteria. Addition of cyclosporin A (CsA) which specifically inhibits T cell receptor-mediated but not cytokine mediated lymphocyte activation [9],[11],[25] showed this response was made up of both innate and adaptive IFN-γ responses. To further define the adaptive IFN-γ component, we compared the frequency of CsA sensitive IFN-γ producing cells against the antibody titer of each individual. Serological evidence of exposure to *B. pseudomallei* is clinically determined by an indirect hemagglutination (IHA) assay which mostly detects antibodies to conserved lipopolysaccharides and/or capsular polysaccharides and is useful in diagnosis of melioidosis particularly in non or low endemic areas [1]. Although the threshold IHA titer for serodiagnosis varies in different countries, in the Northeast Thai population; an IHA titer 1∶40 is considered to be indicative of previous exposure to *B. pseudomallei* in healthy individuals [15],[18]. The frequency of specific, *B. pseudomallei* induced IFN-γ cells closely correlated with the serological status of the donor, whereas no such correlation was observed with control antigens derived from viruses known to be prevalent in the Thai population. Thus environmental exposure to *B. pseudomallei* induces concordant adaptive T and B cell responses as also seen in other examples of infection or vaccination [26],[27].

In mice, IFN-γ is critical for survival of the infected host and NK cells, as well as both CD4^+^ and CD8^+^ T cells contribute to its production [9],[10]. Using intracellular cytokine staining and specific cell depletion we found a similar situation in humans in which all three cell types produced IFN-γ in vitro, with their relative contribution differing according to the serological status of the host. An initial finding was that human NK cells were prominent producers of IFN-γ in vitro, providing some 80% of the IFN-γ positive cells in the first few hours of the culture period. This response was observed in both seronegative and seropositive individuals, was not inhibited by CsA and most likely represents an innate response to the bacterium presumably driven via the generation of IFN-γ inducing cytokines such as IL-12, IL-15 and IL-18 in culture [9],[28]. This observation may also explain the previous findings by Lauw et al of a rapid IFN-γ dependent induction of the chemokines Mig and IP-10 in whole blood cultures of healthy individuals in the presence of dead *B. pseudomallei* [29].

In seropositive individuals, this innate response was supplemented by the presence of IFN-γ positive CD4^+^ T cells and CD8^+^ T cells in both recovered melioidosis patients and asymptomatic healthy control subjects. A predominance of CD4^+^ T cells was observed from the peripheral blood of recovered melioidosis subjects. Haque A, *et al.* also reported that antigen-specific CD4^+^ T cells were important for the resistance against *B. pseudomallei* during the later phase of primary infection [10]. Clear evidence of priming of CD8^+^ T cells was also observed, presumably reflecting the cytoplasmic habitat of the bacterium within host cells [14]. These antigen specific T cells provided the majority of the total IFN-γ generated in culture as evidenced by their higher mean fluorescent intensities (MFI) of IFN-γ staining (Figure S3), larger ELISPOT sizes (data not shown) and by the significant effect of T cell depletion on the IFN-γ ELISPOT response. Of note, we have compared T cell responses by the production of IFN-γ vs. granzyme B by ELISPOT and the results showed high correlation of these 2 indicators in response to *B. pseudomallei* suggesting the importance of cytotoxic T cell response in melioidosis (Figure S4). However, the role of these cells to combat this intracellular pathogen requires further studies.

We then used differential expression of CD45RA and CCR7 to characterize the IFN-γ producing T cells as either central memory (CM), effector memory (EM) or a more recently described effector memory RA (EMRA) populations [30]–[32]. By these criteria, >80% of IFN-γ^+^ CD4^+^ T cells and >90% of IFN-γ^+^ CD8^+^ T cells reacting to *B. pseudomallei* were T_EMRA_ cells, with the remaining minority being T_CM_ and T_EM_ cells. Thus, *B. pseudomallei* predominately induces ‘effector memory RA’ T cells in the peripheral blood that respond rapidly to repeated exposure to the microorganism as also reported with other pathogens such as human cytomegalovirus and human immunodeficiency virus [31],[33]. There was a trend towards a greater contribution of T_EM_ and T_CM_ cells in patients with a history of septicemia compared to localized melioidosis but this did not attain statistical significance and further studies using larger cohort sizes are needed to confirm this.

Several earlier reports established that exposure to *B. pseudomallei* primed human T cells for proliferation and secretion of the macrophage activating cytokine IFN-γ in vitro. However, these studies were restricted to small numbers of individuals in Northern Australia and Papua New Guinea and did not define the frequencies, memory phenotypes of the responding cell populations or the antigen specificity of these responses. The results presented here confirm and extend these findings to a larger sample size in the endemic region of Thailand. A consistent finding in all studies are that T cell responses were greater in seropositive versus seronegative individuals. With the larger group sizes provided in the Thai population we can go further and show that this also correlates with antibody titer, and not simply between antibody positive versus negative status. What is less clear is the relative strength of the cell mediated responses between seropositive healthy donors and recovered patients. Barnes *et al* found that lymphocyte proliferation and IFN-γ production was greater in seropositive healthy donors (n = 8) than those recovered from infection (n = 5), arguing as in the case of tuberculosis, of impaired immunity in those who experienced clinical disease [13]. However, our results in the Thai population showed no difference in the frequencies of IFN-γ producing cells in the recovered melioidosis group versus seropositive healthy donors, although both were clearly greater than seronegative control subjects. In contrast, the amount of IFN-γ secreted (as determined by ELISA) and the frequency of high IFN-γ responders was greater in the recovered group suggesting an increased immune priming following a significant bacterial burden compared to healthy exposed control subjects. Of note, even with the larger sample sizes used here, IFN-γ responses were similar between individuals with and without diabetes, in patients with septicemic versus localized disease or in cases of recurrent versus single episodes of disease [4].

Given the high mortality of acute melioidosis and the problems of treatment, the development of an effective vaccine is an important but difficult task. This is needed to protect individuals living in endemic areas as well as in situations of accidental or purposeful exposure following a bioterrorism scenario. Experimental strategies using wild type bacteria of reduced virulence [34], live attenuated mutants of *B. pseudomallei* [23],[35] and killed whole cells [12] have all been attempted with varying success. However, one important approach requires identification of individual *B. pseudomallei* specific proteins, which are both immunogenic and protective, for inclusion in protein and/or polysaccharide sub-unit based vaccines [36]–[38]. To date, the number of *B. pseudomallei* proteins which have been defined as T cell immunogens in mice or humans is very limited [38]–[40]. In other pathogenic bacteria, ABC transporter proteins have roles in bacterial survival, virulence and pathogenicity, are immunogenic in humans and an increasing number are being considered as candidate vaccine antigens [41]–[43]. We have previously shown that three members of the bacterial ABC transporter family, LolC, PotF and OppA are immunogenic in mice and particularly in the case of LolC provide at least partial protection against lethal challenge with *B. pseudomallei* following immunization in adjuvant [17]. We now show that all three proteins are recognized by T cells from seropositive individuals and could be considered for future vaccine development. No T cell response was observed in *B. pseudomallei* seronegative individuals, arguing that these antigens are relatively specific for *B. pseudomallei* and priming is not the result of cross reactivity against other common bacterial infections in the community.

In conclusion, we provide here the most extensive study to date of the human cell mediated immune response to *B. pseudomallei* and the first to define this aspect of immunity in Thailand, the major endemic focus of melioidosis in the world. Our data demonstrate that *B. pseudomallei* specific CD4^+^ T cells secreting IFN-γ are generated following exposure to the bacterium in the environment and the magnitude of this cellular response correlates with the serological status of the individual. Our findings that NK cells and CD8^+^ T cells also provide a potential source of IFN-γ, may help to explain the apparent lack of impact of HIV/AIDS on the incidence of melioidosis in Thailand. Our ability to detect specific T cell responses to defined *B. pseudomallei* proteins in large numbers of individuals now provides the opportunity to screen candidate antigens for inclusion in protein or protein-polysaccharide conjugate subunit vaccines against this important and emerging infection.

## Supporting Information

Alternative Language Abstract S1 Translation of the Abstract into Thai by Jirawan Mahawantung and Ganjana Lertmemongkolchai
(0.09 MB PDF)

Figure S1 Quantification of IFN-γ production from recovered melioidosis cases vs. seropositive healthy control subjects in response to *B. pseudomallei* in vitro. Whole blood samples from 14 seronegative (S−), 29 seropositive (S+) healthy and 29 recovered melioidosis (R) individuals were incubated with medium alone, 1×10^6^ CFU/ml whole *B. pseudomallei*, 10 ng/ml IL-12+IL-15 and 1.25 µg/ml PHA for 42 hours and collected cultured supernatants for quantitative IFN-γ analysis by ELISA. Horizontal lines indicate mean±SE values of the group, * *P*<0.05, ns-non significant (unpaired t-test).
(0.10 MB PDF)

Figure S2 Immune cell depletion and specific IFN-γ secreting spots in response to hkBp and its ABC transporter proteins. CD3, CD4 and/or CD8 cells were depleted from PBMCs of a seropositive healthy donor by immunomagnetic beads prior to stimulation (ELISPOT details as in Figure 1). Percentage (%) of specific (CsA sensitive) IFN-γ response after depletion was compared to the response of total PBMCs. Data show mean±S.E.
(0.09 MB PDF)

Figure S3 Mean fluorescent intensities of IFN-γ produced by CD3^+^ vs. NK cells in response to *B. pseudomallei*. Whole blood samples of 4 recovered melioidosis were incubated with hkBp for 12 hours and stained for intracellular IFN-γ vs. immune cell surface markers, details as in Figure 4. (A) The mean fluorescent intensity of IFN-γ staining cells analyzed by histograms of medium (grey) overlayered with hkBp (black line) stimulated CD3^+^ vs. NK cells, (B) distribution and mean±S.E of MFI of 4 recovered melioidosis (panel B). * *P*<0.05 (paired t-test).
(0.08 MB PDF)

Figure S4 Correlation of IFN-γ vs. granzyme B production by specific T cells in responses to whole *B. pseudomallei* and three ABC transporter proteins. PBMCs from 54 healthy blood donors were determined for IFN-γ vs. granzyme B by ELISPOT as described in Figure 1 (*r^2^* = correlation coefficient).
(0.10 MB PDF)
