# Supplementary material for: Phenotypic and Functional Characterization of Human Memory T Cell Responses to Burkholderia pseudomallei
Source: PLoS Negl Trop Dis. 2009 Apr 7;3(4):e407. doi: 10.1371/journal.pntd.0000407 (PMC2660609; doi:10.1371/journal.pntd.0000407)
Supplement: Alternative Language Abstract S1 — Translation of the Abstract into Thai by Jirawan Mahawantung and Ganjana Lertmemongkolchai (0.09 MB PDF) [file pntd.0000407.s001.pdf]

ลักษณะและหน้าที่ในการตอบสนองทางภูมิคุ้มกันของทีเซลล์ต่อเชื้อ เบอร์โคลเดอเรีย ปีสูโดมาลิไอ

บทคัดย่อ

พื้นฐานเรื่อง การติดเชื้อแบคทีเรียแกรมลบ เบอร์โคลเดอเรีย ปีสูโดมาลิไอ เป็นสาเหตุสำคัญของการติดเชื้อเมลิออยโดสิสในกระแสเลือดจากชุมชนที่พบมากในภูมิภาคเอเชียตะวันออกเฉียงใต้และภาคเหนือของทวีปออสเตรเลีย และยังมีรายงานพบมากขึ้นในพื้นที่อื่นๆ การศึกษาในสัตว์ทดลองพบว่าการสร้างสารอินเตอร์เฟียรอนแกมมา มีความสำคัญอย่างยิ่งในการต่อต้านการติดเชื้อ เบอร์โคลเดอเรีย ปีสูโดมาลิไอ ส่วนการศึกษาในคนเกี่ยวกับลักษณะการสร้างสารอินเตอร์เฟียรอนแกมมา และการตอบสนองต่อแอนติเจนจากเชื้อยังมีรายงานน้อยมาก

วิธีการ: เก็บตัวอย่างเลือดจากอาสาสมัครสุขภาพดีที่อาศัยในพื้นที่ระบาดของเชื้อจำนวน ๑๓๓ รายและผู้ป่วยที่เคยเป็นโรคติดเชื้อเมลิออยโดสิสจำนวน ๖๐ ราย พร้อมกับกลุ่มควบคุมจำนวน ๓๑ ราย นำมาทดสอบการกระตุ้นด้วยตัวเชื้อและโปรตีนของเชื้อในหลอดทดลอง พร้อมทั้งตรวจวัดการสร้างสารอินเตอร์เฟียรอนแกมมา โดยใช้เทคนิคอีไลซ่าและโฟลไซโตมิเตอร์

ผลการทดลอง พบว่าเบอร์โคลเดอเรีย ปีสูโดมาลิไอ สามารถกระตุ้นการสร้างสารอินเตอร์เฟียรอนแกมมาอย่างสูงตามแบบธรรมชาติ นอกจากนี้ยังพบว่าในกลุ่มอาสาสมัครสุขภาพดีที่มีแอนติบอดีซึ่งแสดงว่าเคยสัมผัสกับเชื้อ และกลุ่มผู้ที่เคยป่วยด้วยเชื้อชนิดนี้สามารถสร้างสารนี้ผ่านทางทีเซลล์ชนิดซีดี ๔ และซีดี ๘ ที่ทำงานตอบสนองต่อตัวเชื้อและสารโปรตีนของเชื้อ ได้แก่ โลล์ซี ออปเอ และพอทเอฟ ซึ่งอยู่ในตระกูลเอบีซี ทรานส์ปอร์ตเตอร์ โดยการตอบสนองทางภูมิคุ้มกันนี้ยังมีลักษณะการจดจำส่วนใหญ่เป็นทีเซลล์ชนิดอีเอ็มอาร์เอ และยังพบสัมพันธ์กับปริมาณแอนติบอดี

สรุป บุคคลที่มีถิ่นพำนักในพื้นที่ระบาดของโรคติดเชื้อเมลิออยโดสิสสามารถแสดงการตอบสนองทางภูมิคุ้มกันได้อย่างชัดเจนผ่านทางทีเซลล์ โดยสร้างสารอินเตอร์เฟียรอนแกมมาที่สัมพันธ์กับแอนติบอดี ซึ่งความสามารถในการตอบสนองของทีเซลล์ต่อสารโปรตีนของเชื้อชนิดนี้ ทำให้สามารถนำมาใช้คัดเลือกส่วนที่จะนำไปพัฒนาเป็นวัคซีนต่อไปโดยใช้ทั้งชนิดโปรตีนย่อยหรือมีองค์ประกอบสารคาร์โบไฮเดรด เพื่อเป็นการป้องกันการเกิดโรคติดเชื้อที่มีความสำคัญอย่างยิ่งแต่ถูกละเลยนี้
