# Supplementary figures and images for: Phenotypic and Functional Characterization of Human Memory T Cell Responses to Burkholderia pseudomallei
Source: PLoS Negl Trop Dis. 2009 Apr 7;3(4):e407. doi: 10.1371/journal.pntd.0000407 (PMC2660609; doi:10.1371/journal.pntd.0000407)

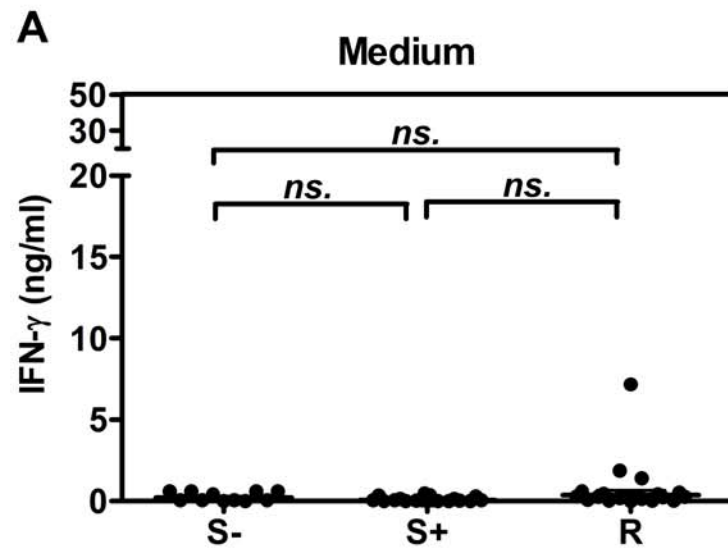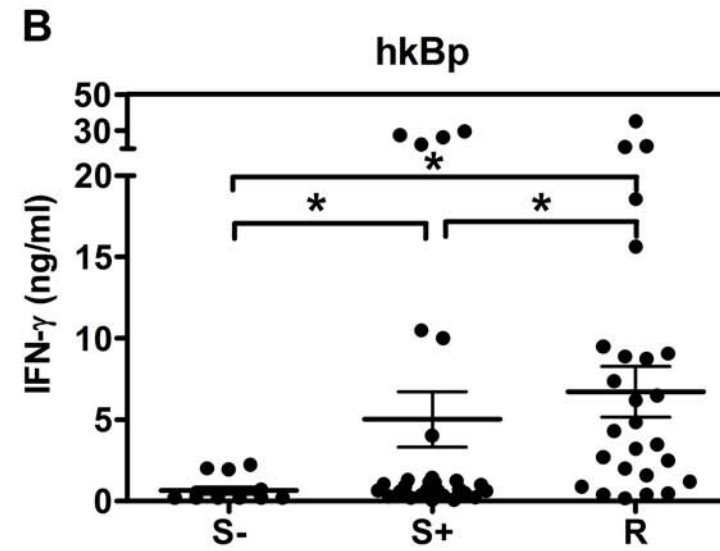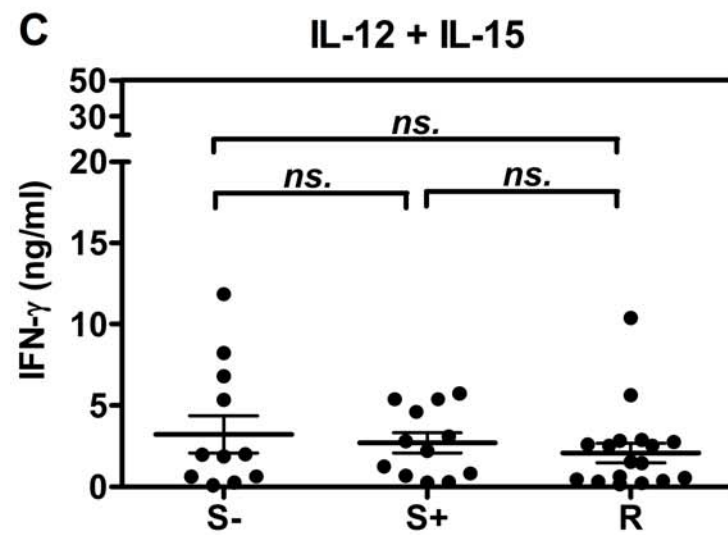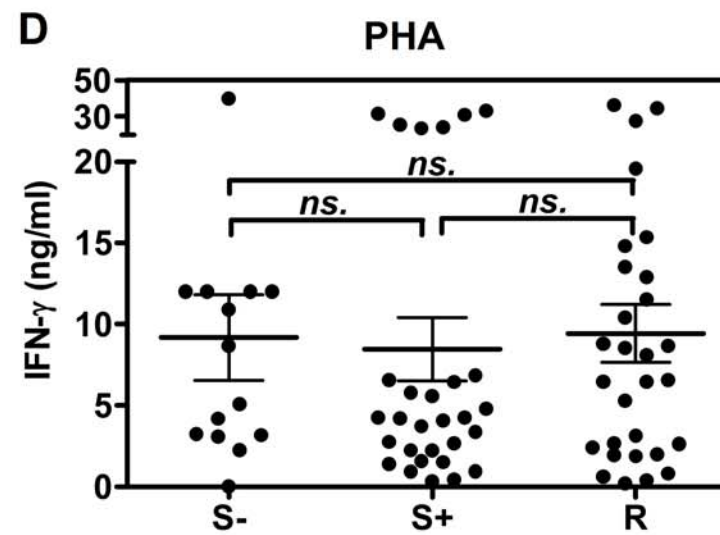

Supplement: Figure S1 — Quantification of IFN-γ production from recovered melioidosis cases vs. seropositive healthy control subjects in response to B. pseudomallei in vitro. Whole blood samples from 14 seronegative (S−), 29 seropositive (S+) healthy and 29 recovered melioidosis (R) individuals were incubated with medium alone, 1×10^6^ CFU/ml whole B. pseudomallei, 10 ng/ml IL-12+IL-15 and 1.25 µg/ml PHA for 42 hours and collected cultured supernatants for quantitative IFN-γ analysis by ELISA. Horizontal lines indicate mean±SE values of the group, * P<0.05, ns-non significant (unpaired t-test). (0.10 MB PDF) [file pntd.0000407.s002.pdf]

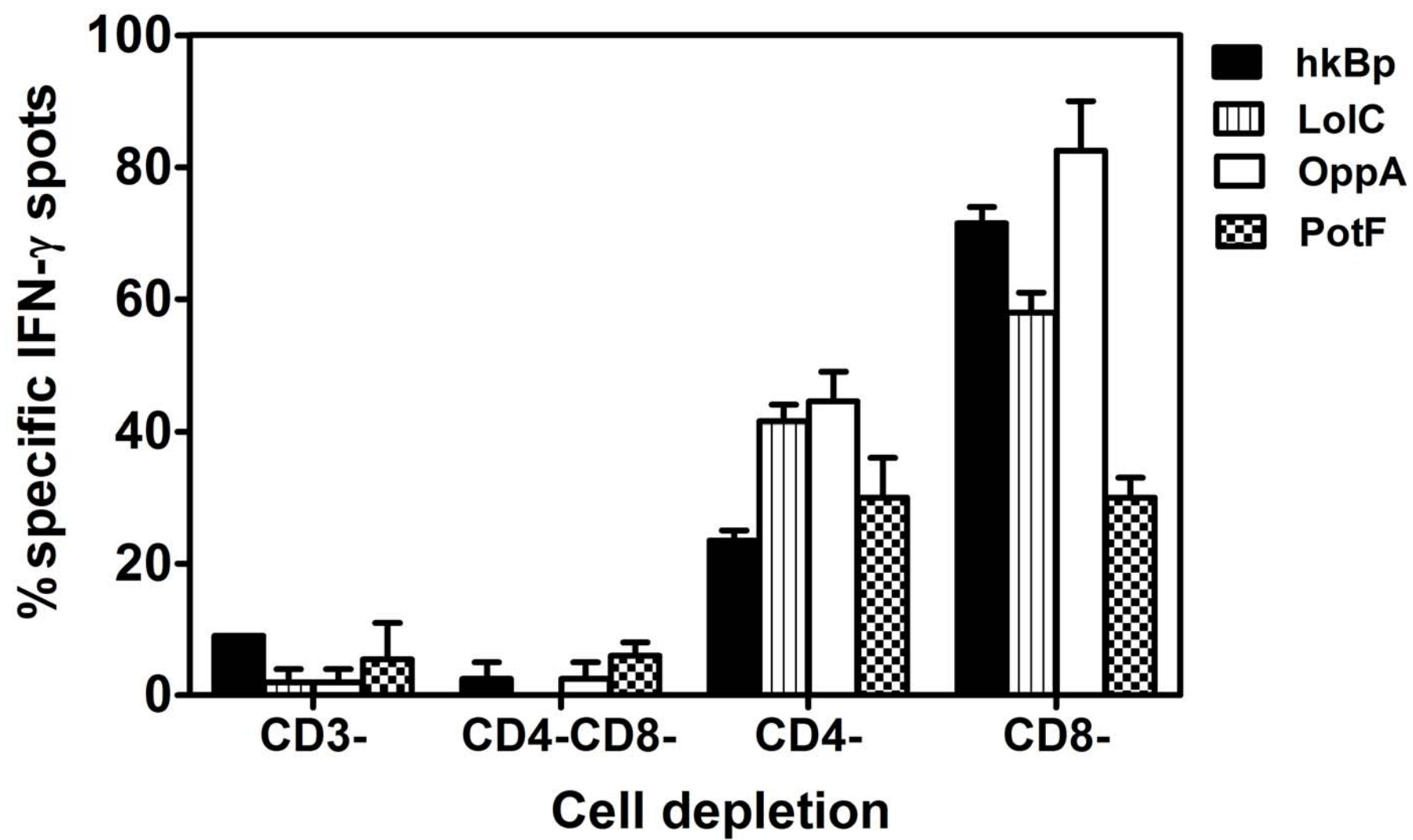

Supplement: Figure S2 — Immune cell depletion and specific IFN-γ secreting spots in response to hkBp and its ABC transporter proteins. CD3, CD4 and/or CD8 cells were depleted from PBMCs of a seropositive healthy donor by immunomagnetic beads prior to stimulation (ELISPOT details as in Figure 1). Percentage (%) of specific (CsA sensitive) IFN-γ response after depletion was compared to the response of total PBMCs. Data show mean±S.E. (0.09 MB PDF) [file pntd.0000407.s003.pdf]

**A**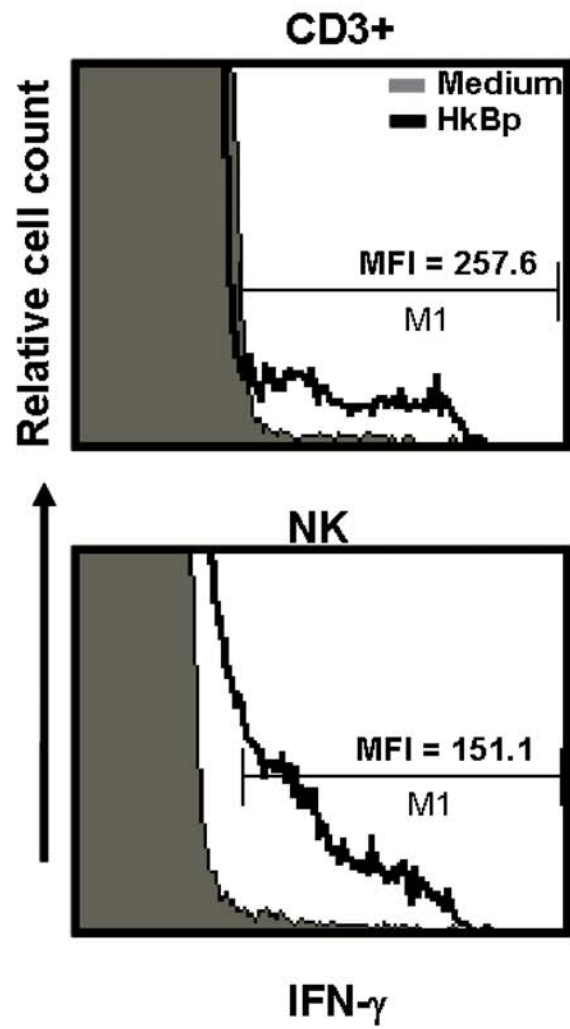**B**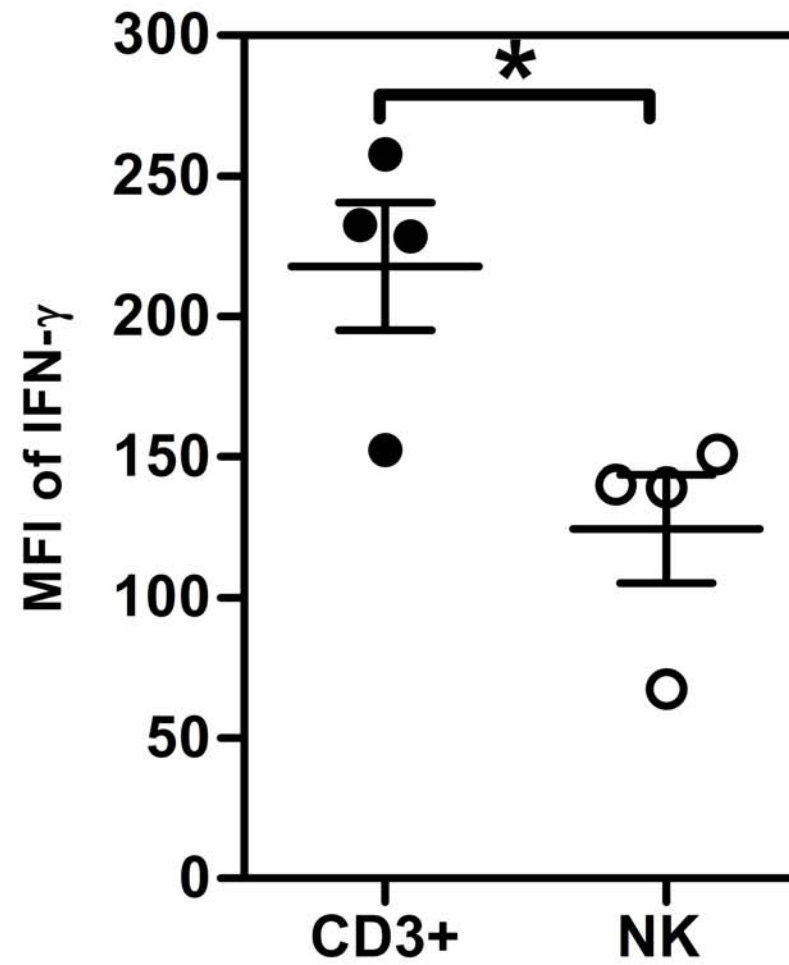

Supplement: Figure S3 — Mean fluorescent intensities of IFN-γ produced by CD3+ vs. NK cells in response to B. pseudomallei. Whole blood samples of 4 recovered melioidosis were incubated with hkBp for 12 hours and stained for intracellular IFN-γ vs. immune cell surface markers, details as in Figure 4. (A) The mean fluorescent intensity of IFN-γ staining cells analyzed by histograms of medium (grey) overlayered with hkBp (black line) stimulated CD3+ vs. NK cells, (B) distribution and mean±S.E of MFI of 4 recovered melioidosis (panel B). * P<0.05 (paired t-test). (0.08 MB PDF) [file pntd.0000407.s004.pdf]

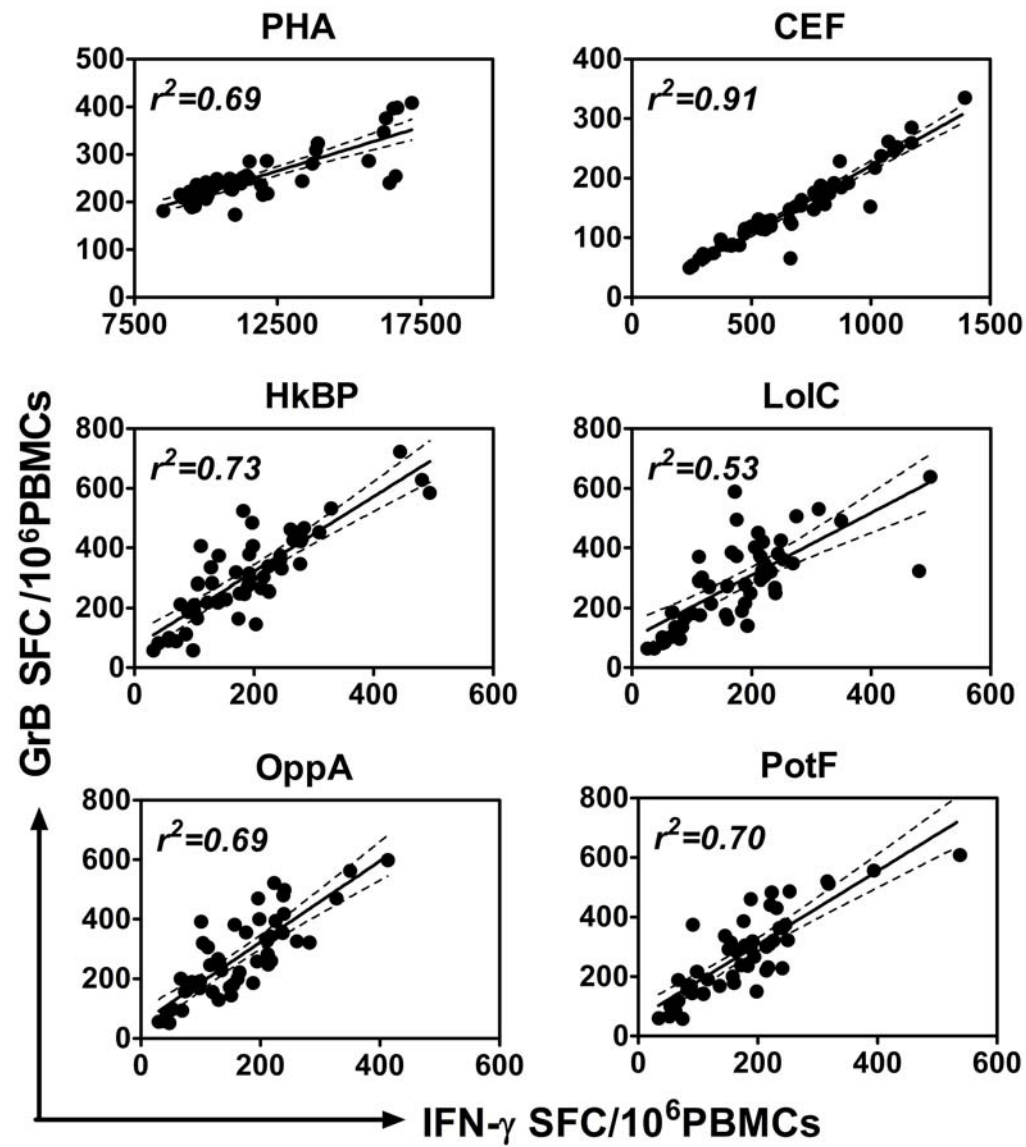

Supplement: Figure S4 — Correlation of IFN-γ vs. granzyme B production by specific T cells in responses to whole B. pseudomallei and three ABC transporter proteins. PBMCs from 54 healthy blood donors were determined for IFN-γ vs. granzyme B by ELISPOT as described in Figure 1 (r2 = correlation coefficient). (0.10 MB PDF) [file pntd.0000407.s005.pdf]
